# Supplementation with paraformic acid in the diet improved intestinal development through modulating intestinal inflammation and microbiota in broiler chickens

**DOI:** 10.3389/fmicb.2022.975056

**Published:** 2022-09-20

**Authors:** Junwei Li, Yang Liu, Jiaxing Niu, Changwei Jing, Ning Jiao, Libo Huang, Shuzhen Jiang, Lei Yan, Weiren Yang, Yang Li

**Affiliations:** ^1^Shandong Provincial Key Laboratory of Animal Biotechnology and Disease Control and Prevention, College of Animal Science and Veterinary Medicine, Shandong Agricultural University, Tai’an, China; ^2^Qingdao Huaxin Feed Co., Ltd., Qingdao, China; ^3^Technical Department, Shandong Chinwhiz Co., Ltd., Weifang, China; ^4^Shandong New Hope Liuhe Group Co., Ltd., Qingdao, China

**Keywords:** chickens, inflammation, microbiota, organic acid, small intestine

## Abstract

The aim of this study was to explore the effects of supplementing paraformic acid (PFA) to the diet of broiler chickens on intestinal development, inflammation, and microbiota. A total of 378 healthy 1-day-old Arbor Acres broilers with similar birth weight were used in this study, and randomly assigned into two treatment groups. The broiler chickens were received a basal diet or a basal diet supplemented with 1,000 mg/kg PFA. Results showed that PFA supplementation increased (*P* < 0.05) small intestinal villus height and villus height/crypt depth ratio, elevated intestinal mucosal factors (mucin 2, trefoil factor family, and zonula occludens-1) concentrations, and upregulated mNRA expression of y + L amino acid transporter 1. Moreover, PFA supplementation decreased (*P* < 0.05) the concentrations of inflammatory cytokines (tumor necrosis factor-alpha, interleukin-1beta, interleukin-6, and interleukin-10), activities of caspase-3 and caspase-8, and mNRA expressions of Toll-like Receptor 4, nuclear factor-kappa B, *Bax*, and *Bax*/*Bcl-2* ratio in small intestinal mucosa. Dietary PFA supplementation also increased (*P* < 0.05) alpha diversity of cecal microbiota and relative abundance of *Alistipes*. The present study demonstrated that supplementation of 1,000 mg/kg PFA showed beneficial effects in improving intestinal development, which might be attributed to the suppression of intestinal inflammation and change of gut microbiota composition in broiler chickens. These findings will aid in our knowledge of the mechanisms through which dietary PFA modulates gut development, as well as support the use of PFA in poultry industry.

## Introduction

A healthy and well-developed gut is essential for nutrient absorption and serves as a necessary barrier against pathogen invasion (Kogut et al., 2017). However, with the rapid expansion of the scale of intensive farming worldwide, poultry production faces increasing challenges, such as pathogenic bacteria, environmental variables, and feed hygiene, which raise the risks of intestinal diseases in broiler chickens (Qian et al., 2018; Caekebeke et al., 2020). Previous studies have demonstrated that intestinal diseases can limit intestinal development, cause dysfunction of intestinal digestion and absorption, and induce an intestinal flora imbalance, resulting in growth restriction, disease, and even mortality in broiler chickens (Roberts et al., 2015; Shi et al., 2018). For many years, the subtherapeutic use of antibiotic growth promoters (AGP) has been an economically viable means of enhancing animal performance (Bedford, 2000). With the prohibition of AGP due to its significant negative effects on environmental conditions and human health, it is critical to find new feed additives that promote intestinal health and development in poultry production (Castanon, 2007; Muaz et al., 2018).

Acidifiers such as pure organic acids have been used as feed preservatives for decades to prevent microbial and fungal destruction of feedstuffs (Hu et al., 2019; Zhang et al., 2019; Abd El-Hack et al., 2022). Growing studies suggested that organic acids could be used as powerful tool in maintaining gut health by suppressing the proliferation of acid intolerance bacteria, such as *E. coli* and *Clostridium perfringens*, leading to improvement of growth performance in poultry (Gharib et al., 2012; Ateya et al., 2019; Hu et al., 2019). It was reported that dietary supplementation with formic acid (FA) benefited to growth performance, immune function, intestinal development, and microbiological characteristics of broilers (Hernández et al., 2006; Garcia et al., 2007; Ragaa and Korany, 2016). However, FA has a strong, pungent odor and corrosiveness to gastrointestinal tract, which limit its usage in animal husbandry (Luise et al., 2020). Recently, FA salts and its derivatives, such as calcium formate, benzoic acid, and potassium diformate, have deserved more and more attention in poultry production due to their little or no corrosive effect and also efficient against harmful microorganisms (Izat et al., 1990; Józefiak et al., 2010; Ragaa and Korany, 2016). Paraformic acid (PFA) is a hyperpolymer formed by dehydration polymerization between two FA molecules. It has not been reported that whether PFA can promote intestinal development of broiler chickens.

Therefore, the aim of this study was to explore the effects of supplementing PFA to the diet of broiler chickens on intestinal development, and provide a reference for the use of PFA in poultry industry.

## Materials and methods

### Animals and diets

A total of 378 healthy 1-day-old Arbor Acres broilers with similar birth weight (48.47 ± 0.43 g) were used in this study. All broilers were randomly assigned into two treatment groups with seven replicates of 27 broiler chickens each and housed in three-level wired cages placed in a light- and temperature-controlled room with constant illumination in a 42-day study. The treatment groups were as follows: broiler chickens received a basal diet (CON group), or broiler chickens received a basal diet supplemented with 1,000 mg/kg PFA (PFA group). The basal diets (Supplementary Table 1) were formulated based on nutrient requirements of the National Research Council [NAC] (1994), and PFA was provided by Omega Nutrition Group (Spain) & Numega Nutrition Pte. Ltd. The broilers were fed according to a two-phase feeding program (0–21 day and 21–42 day), and had free access to feed and water throughout the experiment. A Newcastle disease vaccine and an inactivated infectious bursal disease vaccine were inoculated on days 7 and 14 of the trial, respectively. The temperature of the room was maintained at 35°C at the first week, and then gradually reduced to 21°C at the rate of 0.5°C daily.

### Sample collection

On day 42 of experiment, one broiler from each replicate (7 birds per group) with similar body weight (BW) to the cage average were selected to collect intestinal tissue samples after being narcotized by CO_2_ asphyxiation. Intestinal segments with a length of 2 cm were cut from the medium of small intestine, and fixed in 4% paraformaldehyde for 24 h after being flushed gently with a 0.9% saline solution. Then mucosal tissue was carefully scraped with a sterile glass slide from the medium of small intestine that had been washed using ice-cold saline solution, and subsequently stored at −80°C after being chilled in liquid nitrogen. Besides, the cecal contents were collected and placed in sterile bags immediately, and stored at -80°C for microbiological analysis.

### Measurements of intestinal morphology

The fixed intestinal segments were dehydrated in ethanol and xylene solutions, and embedded according to conventional paraffin-embedding protocol, followed by being cut into 5-μm thin slices using Leica semi-automatic microtome (Leica Co., Wetzlar, Germany). Then the slices were processed by hematoxylin and eosin staining. The intestinal morphology was examined, and villus height (VH), crypt depth (CD) and VH/CD ratio were analyzed according to the method described in Chen et al. (2021).

### Determination of intestinal mucosal barrier factors and total protein concentrations

Intestinal mucosal barrier factors including mucin 2 (MUC2), trefoil factor family (TFF), transforming growth factor-α (TGF-α), and zonula occludens-1 (ZO-1) were determined with the specific ELISA kits (Jiangsu Meimian Industrial Co., Ltd., Jiangsu, China), following the manufacturer’s instructions strictly. Briefly, the supernatants of intestinal mucosa samples were harvested after being homogenized in ice-cold saline solution (1:9, wt/vol) and centrifugated at 12,000 × *g* for 15 min. Then 50 μl of diluted standard solutions and supernatants were added to the prepared microplates, respectively, and incubated 30 min at 37°C after being sealed with microplate sealers. The HRP-Conjugate reagent (50 μl) was added to each well after the microplate was washed five times with Wash Buffer, and the microplate was incubated 30 min at 37°C again. After being washed five times again, the wells were added with Chromogenic solution A and B (50 μl each) in order, and the microplate was allowed to stand at 37°C for 10 min under dark condition after gently shaking. Finally, the optical density of each well was determined in 15 min using a microplate reader set at 450 nm, following addition of Stop Solution (50 μl). The concentrations of MUC2, TFF, TGF-α, and ZO-1 were normalized to each sample’s total protein concentration which was quantified with a BCA protein assay (Jiangsu Meimian Industrial Co., Ltd.) (Lang et al., 2022).

### Determination of intestinal mucosal caspases activities

Intestinal mucosal activities of caspase-3, caspase-8, and caspase-9 were determined with the specific ELIAS kits purchased from Beyotime Biotech (Shanghai, China) as descried previously (Chen et al., 2021), and normalized to each sample’s total protein concentration.

### Determination of intestinal mucosal inflammatory cytokines and secretory immunoglobulin A concentrations

The intestinal mucosal concentrations of tumor necrosis factor-alpha (TNF-α), interleukin-1beta (IL-1β), interleukin-6 (IL-6), interleukin-10 (IL-10), interferon-γ (IFN-γ), and secretory immunoglobulin A (SIgA) were examined with ELISA kits (R&D Systems Inc., Minneapolis, MN, United States) according to the detection steps of ELISA operation described in Chen et al. (2021). Inflammatory cytokines and SIgA concentrations in intestinal mucosa were normalized to each sample’s total protein concentration.

### Determination of relative mRNA expression in intestinal mucosa

The mRNA expression levels of occludin (*OCLN*), claudin 2 (*CLDN2*), claudin 3 (*CLDN3*), *ZO-1*, glucose transporter 2 (*GLUT2*), Na + /glucose cotransporter (*SGLT1*), y + L amino acid transporter 1 (*y* + *LAT1*), fatty acid binding protein (*FABP1*), cationic amino acid transporter 1 (*CAT1*), Bax, Bcl-2, Toll-like Receptor 4 (*TLR4*), and nuclear factor-kappa B (*NF-*κ*B*) in intestinal mucosa samples were assessed using a CFX-96 real-time PCR detection system (Bio-Rad, Hercules, CA, United States). Primer sequences used for real-time PCR in this study are shown in Supplementary Table 2. The detailed procedure of the relative mRNA expression determination was described in Zhang P. et al. (2021). The β-actin gene was amplified in parallel as the internal control for gene normalization and quantification. The 2^–Δ^ ^Δ^ ^Ct^ method was used to calculate the relative mRNA abundances of target genes in intestinal mucosa samples. All samples were measured in triplicate, and product sizes and quantities were determined by agarose gel electrophoresis.

### Determination of pH values of cecal digesta

The determination of pH values of cecal digesta was conducted as previously described in Li et al. (2020) using the pH meter (PHS-3C PH, Shanghai, China).

### Microbial analysis

Total genomic DNA was isolated from cecal digesta by the Omega Bio-tek E.Z.N.A. ™ stool DNA kit (Norcross, GA, United States), followed by DNA concentration and purity examination by agarose gel electrophoresis (Chen et al., 2021). Briefly, the V4 hypervariable region of 16S rDNA was amplified by using 515F and 806R primer (Li et al., 2019). The produced library quality was examined using a Qubit 2.0 Fluorometer (Thermo Fisher Scientific, United Kingdom). Sequencing of the library was carried out on the Illumina HiSeq PE2500 platform at the Novogene Bioinformatics Technology Co., Ltd. (Beijing, China), after which 250 bp paired-end sequences were generated. Paired-end sequences were merged using FLASH (v1.2.7) (Magoè and Salzberg, 2011), and the chimera sequences were removed through comparing with the Silva database using UCHIME algorithm to obtain the effective sequences after quality filtering on the raw tags (Edgar et al., 2011). Sequences were clustered into operational taxonomic units (OTUs) at least 97% sequence similarity using Uparse software (Edgar, 2013), and taxonomic information was annotated using the Silva Database based on Mothur algorithm (Quast et al., 2013). Alpha diversity (including observed species, Shannon index, Simpson index, ACE index, and Chao 1 index) and beta diversity were used to analyze complexity of species diversity for a sample and differences of samples in species complexity, respectively. Principal Coordinate Analysis (PCoA) was performed based on bray-curtis distances to visualize the dissimilarity matrices of OTUs, and the analysis of similarity (ANOSIM) was applied to examine the significant differences among the microbial communities.

### Statistical analyses

The individual chicken data were used to access the effects on all variables. Statistical analyses for all data were performed with *t*-test of SAS (9.4 Inst. Inc., Cary, NC, United States). The Shapiro-Wilk W statistic was applied to check normality of the data, and the data that were not normally distributed were transformed to achieve approximated normality. Spearman’s correlation and linear regression analysis were used to assess the associations between bacterial abundance and intestinal mucosal concentrations of immunological markers, as well as between microorganisms. Values are expressed as mean ± standard error. Statistical significance was set at *P* < 0.05, and a trend toward significance was considered at 0.05 ≤ *P* < 0.10.

## Results

### Intestinal morphology

**FIGURE 1:**
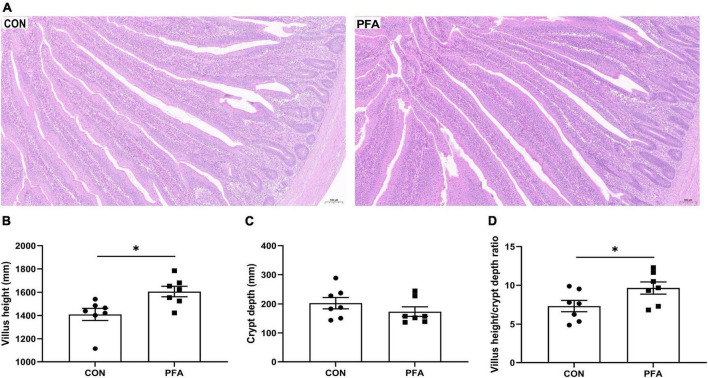
Effect of dietary paraformic acid supplementation on intestinal morphology in broiler chickens. **(A)** Hematoxylin and eosin photomicrographs obtained at 100 × magnification; **(B)** Villus height; **(C)** Crypt depth; **(D)** Villus height/crypt depth ratio. CON, broiler chickens fed basal diet; PFA, broiler chickens fed basal diet supplemented with 1,000 mg/kg paraformic acid. Values are mean ± standard error (*n* = 7). Differences between treatments were displayed by *P < 0.05.

The effects of PFA supplementation on intestinal morphology are presented in Figure 1. Compared with CON group, the villus in PFA group showed thicker and denser (Figure 1A). Broilers fed the diet supplemented with PFA had significantly higher (*P* < 0.05) intestinal VH (Figure 1B) and VH/CD ratio (Figure 1D) than broilers fed the CON diet. There was no significant difference (*P* > 0.05) in intestinal CD (Figure 1C) between broilers fed the CON diet and the PFA diet.

### Intestinal mucosal barrier functions, apoptosis regulators, and inflammatory factors concentrations

**FIGURE 2:**
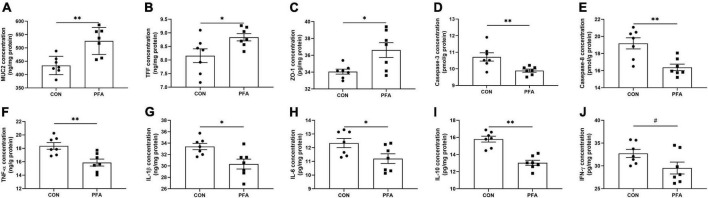
Effects of dietary paraformic acid supplementation on intestinal mucosal barrier functions, apoptosis regulators, and inflammatory factors concentrations in broiler chickens. **(A)** Mucin (MUC2); **(B)** Trefoil factor family (TFF); **(C)** Zonula occludens-1 (ZO-1); **(D)** Caspase-3; **(E)** Caspase-9; **(F)** Tumor necrosis factor-alpha (TNF-α); **(G)** Interleukin-1beta (IL-1β); **(H)** Interleukin-6 (IL-6); **(I)** Interleukin-10 (IL-10); **(J)** Interferon-γ (IFN-γ). CON, broiler chickens fed basal diet; PFA, broiler chickens fed basal diet supplemented with 1,000 mg/kg paraformic acid. Values are mean ± standard error (*n* = 7). Differences between treatments were displayed by ^#^0.05 ≤ *P* < 0.10, *P < 0.05, and ^**^P < 0.01.

Dietary supplementation with 1,000 mg/kg PFA significantly increased (*P* < 0.05) MUC2 (Figure 2A), TFF (Figure 2B), and ZO-1 (Figure 2C) concentrations, and significantly decreased (*P* < 0.05) activities of caspase-3 (Figure 2D) and caspase-8 (Figure 2E) and concentrations of TNF-α (Figure 2F), IL-1β (Figure 2G), IL-6 (Figure 2H), and IL-10 (Figure 2I) in intestinal mucosa of broilers. Besides, PFA supplementation tended to decrease (*P* < 0.10) intestinal mucosal IFN-γ concentration compared with CON group (Figure 2J). There were no significant differences (*P* > 0.05) in TGF-α and SIgA concentrations as well as caspase-9 activity in intestinal mucosa of broilers (Supplementary Figure 1).

### Genes expressions in intestinal mucosa

**FIGURE 3:**
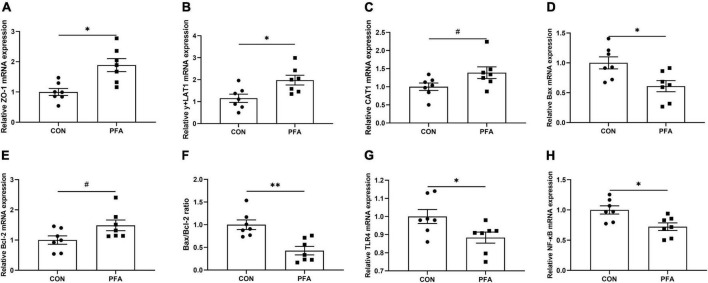
Effect of dietary paraformic acid supplementation on relative mRNA expression in intestinal mucosa of broilers. **(A)** Zonula occludens-1 (ZO-1); **(B)** y + L amino acid transporter 1 (y + LAT1); **(C)** Cationic amino acid transporter 1 (CAT1); **(D)** Bax; **(E)** Bcl-2; **(F)** Bax/Bcl-2 ratio; **(G)** Toll-like Receptor 4 (TLR4); **(H)** Nuclear factor-kappa B (NF-κB). CON, broiler chickens fed basal diet; PFA, broiler chickens fed basal diet supplemented with 1,000 mg/kg paraformic acid. Values are mean ± standard error (*n* = 7). Differences between treatments were displayed by ^#^0.05 ≤ *P* < 0.10, *P < 0.05, and ^**^P < 0.01.

**FIGURE 4:**
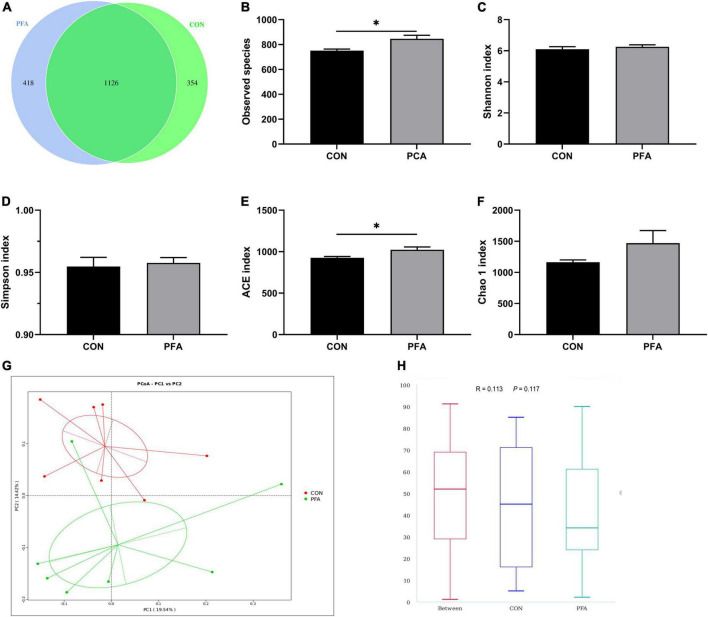
Effect of dietary paraformic acid supplementation on diversity and richness of cecal microbiota in broilers. **(A)** A Venn diagram generated to display the common and unique operational taxonomic units between two groups; **(B)** Observed species; **(C)** Shannon index; **(D)** Simpson index; **(E)** ACE index; **(F)** Chao 1 index; **(G)** The principal coordinate analysis (PCoA) profile of bray_curtis distance; **(H)** Analysis of similarity. CON, broiler chickens fed basal diet; PFA, broiler chickens fed basal diet supplemented with 1,000 mg/kg paraformic acid. Values are mean ± standard error (*n* = 7). Differences between treatments were displayed by *P < 0.05.

As shown in Figure 4, PFA supplementation in broiler diet significantly increased (*P* < 0.05) *ZO-1* (Figure 3A) and *y* + *LAT1* (Figure 3B) mRNA expressions, and tended to increase (*P* < 0.05) *CAT1* (Figure 3C) and *Bcl-2* (Figure 3E) mRNA expression in intestinal mucosa. Besides, PFA group showed significantly lower (*P* < 0.05) mucosal *Bax* (Figure 3D), *TLR4* (Figure 3G) and *NF-*κ*B* (Figure 3H) mRNA expressions as well as *Bax*/*Bcl-2* ratio (Figure 3F). There were no significant differences (*P* > 0.05) in the mRNA expressions of *OCLN*, *CLDN2*, *CLDN3*, *GLUT2*, *SGLT1*, and *FABP1* in intestinal mucosa (Supplementary Figure 2).

### Cecal pH of digesta

Effect of dietary paraformic acid supplementation on pH values of cecal digesta in broiler chickens is shown in Table 1. Broilers in PFA group had significantly lower (*P* < 0.05) cecal pH values than those in CON group.

**TABLE 1:** Effect of dietary paraformic acid supplementation on pH values of cecal digesta in broiler chickens.

Items, %	Treatment^1^		*P*-value
	CON	PFA	
pH value	6.70 ± 0.07	6.43 ± 0.08	0.034

Values are mean ± standard error (*n* = 7). Differences were considered statistically significant when *P* < 0.05.

^1^CON, broilers fed a basal diet; PFA, broilers fed a basal diet supplemented with 1,000 mg/kg paraformic acid.

### Microbial diversity in cecal digesta

As shown in Supplementary Table 3, a total of 825,033 total tags, 778,291 taxon tags, 13 unclassified tags, 56,729 unique tags, and 12,147 OTUs were obtained from 14 cecal digesta samples of two treatment groups. Moreover, the species accumulation curves (Supplementary Figure 3) tend to flatten with analyzed sequences number increasing up to 14, demonstrating that our samples were sufficient for OTU testing and prediction of species richness of samples. The bacteria community diversity and richness are shown in Figure 4. The overall OTUs of cecal bacteria differ between groups, and broilers in PFA group showed an increased number of OTUs (Figure 4A). The two groups shared 1,129 common OTUs. Compared with CON group, PFA group showed significantly higher (*P* < 0.05) observed species (Figure 4B) and ACE index (Figure 4E). No significant differences were observed (*P* > 0.05) in Shannon index (Figure 4C), Simpson index (Figure 4D), and Chao 1 index (Figure 4F). The PCoA plot (Figure 4G) drawn based on the bray_curtis distances revealed that the PFA samples dispersed far apart with the CON samples, and ANOSIM (Figure 4H) showed the two groups had significantly different bacterial community structures (*P* < 0.05).

### Relative abundance of cecal microbiota

The top 10 phyla in relative abundance of cecal microbiota are shown in shown in Supplementary Table 4. The most predominant phyla in cecal samples of broilers are Bacteroidetes and Firmicutes. Dietary PFA supplementation significantly decreased (*P* < 0.05) Halobacterota abundance, and tended to decrease (*P* < 0.10) Campylobacterota abundance.

**FIGURE 5:**
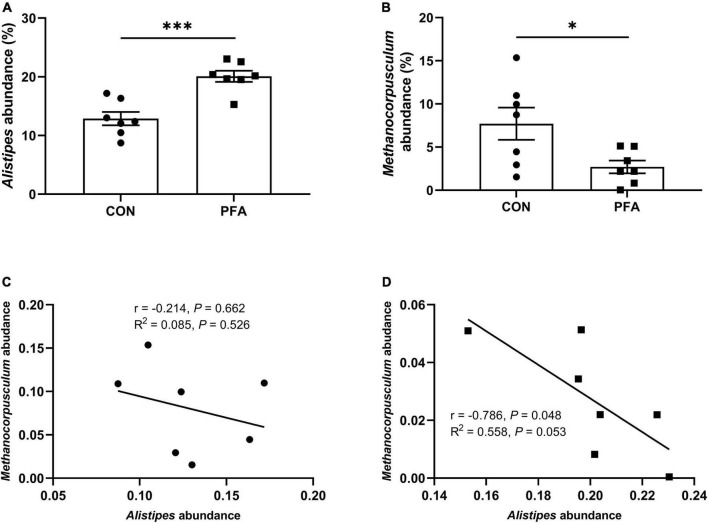
Relative abundance of cecal microbiota (at the genus level) that are significantly different between two treatments in broilers. **(A,B)** Relative abundances of *Alistipes* and *Methanocorpusculum*. Differences between treatments were displayed by *P < 0.05 and ^***^P < 0.001. Values are mean ± standard error. **(C,D)** The correlation between the *Alistipes* and *Methanocorpusculum* abundances for CON group and PFA group based on Spearman’s correlation test and simple linear regression analysis. Statistical significance was set at *P* < 0.05. CON, broiler chickens fed basal diet; PFA, broiler chickens fed basal diet supplemented with 1,000 mg/kg paraformic acid, *n* = 7.

The relative abundance at genus level in broiler cecal microbiota (top 35 genera) in shown in Figure 5 and Supplementary Table 5. The identified most plentiful genera in cecal samples were *Alistipes*, *Bacteroides*, and *Desulfovibrio*. PFA group had significantly higher (*P* < 0.05) *Alistipes* abundance and significantly lower (*P* < 0.05) *Methanocorpusculum* abundance than CON group. Besides, PFA group tended to had lower (*P* < 0.10) abundances of *Helicobacter*, *Christensenellaceae_R-7_group*, and *Ruminococcus* in cecal samples than CON group. In PFA group, the *Alistipes* abundance was significantly negatively (*P* = 0.048) correlated with *Methanocorpusculum* abundance, and the *Methanocorpusculum* abundance tended to (*P* = 0.053) be linearly decreased with the *Alistipes* abundance increasing. No significant correlation (*P* > 0.05) between *Alistipes* abundance and *Methanocorpusculum* abundance was observed in cecal digesta of CON group.

### Spearman’s correlation analysis between *Alistipes* and *Methanocorpusculum* abundances and mucosal immunological markers concentrations

**FIGURE 6:**
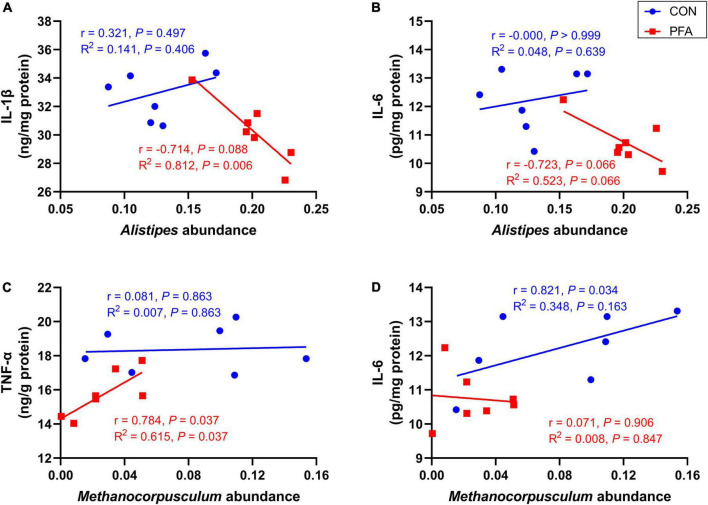
Correlation analysis between *Alistipes* and *Methanocorpusculum* abundances and mucosal immunological markers concentrations. **(A,B)** Correlation analysis between *Alistipes* and interleukin-1beta (IL-1β) and interleukin-6 (IL-6) for CON group and PFA group; **(C,D)** Correlation analysis between *Methanocorpusculum* and tumor necrosis factor-alpha (TNF-α) and IL-6 for CON group and PFA group. Correlation analysis was based on Spearman’s test and simple linear regression. Statistical significance was set at *P* < 0.05, *n* = 7.

As shown in Figure 6 and Supplementary Figure 4, intestinal mucosal concentrations of IL-1β and IL-6 tended to decrease (*P* < 0.10) with increasing relative abundance of *Alistipes* in broilers of PFA group. The relative abundance of *Methanocorpusculum* was significantly positively (*P* < 0.05) correlated with mucosal TNF-α concentration of PFA group and IL-6 concentration of CON group, and intestinal mucosal TNF-α concentration increased linearly (*P* = 0.037) as *Methanocorpusculum* abundance increased in PFA group.

## Discussion

Intestinal morphology and barrier integrity are two crucial indicators of intestinal development (Li et al., 2020; Beumer and Clevers, 2021). In the present study, PFA supplementation in the diet increased VH and VH/CD ratio of small intestine. Previous study also demonstrated that broilers fed the diets supplemented with FA or potassium diformate had higher intestinal VH than those fed a basal diet (Abd El-Hack et al., 2022). The small intestine is the principal organ in charge of nutrient absorption, and the crypt-villus structure is responsible for efficient nutrition intake (Beumer and Clevers, 2021). The VH and VH/CD are related to the digestion and absorption capacity of the intestine (Chen et al., 2021). Consistently, compared with CON group, PFA group had higher intestinal expressions of *y* + *LAT1* and *CAT1* which were responsible for amino acids transport (Closs et al., 2006; del Amo et al., 2008). Garcia et al. (2007) reported that dietary FA supplementation significantly increased the digestibility of crude protein. It might suggest that PFA supplementation was helped to enhance the digestion and absorption capacity of the intestine in this study. Besides, we also found that PFA supplementation increased ZO-1, MUC2, and TFF levels in small intestinal mucosa in the current study. As a regulator of paracellular permeability in epithelia and endothelia, the major component of tight junction protein (TJP) ZO-1 plays an important role in maintaining intestinal barrier function and defensing systemic inflammatory diseases through interacting with the gap, actin cytoskeleton, and adherens junction proteins (Dai et al., 2020; Lai et al., 2021). Trefoil factor family and mucins are typical exocrine products of mucous epithelia. Mucin2, predominantly produced by the goblet cells, is the principal mucin in the small intestine and located throughout the surface of the intestinal epithelium (Liu et al., 2020). Recent researches demonstrated that MUC2 is involved in intestinal barrier protection, microbiome homeostasis regulation, and diseases prevention (Kim and Khan, 2013; Liu et al., 2020). Trefoil factor family peptides are typically co-secreted together with mucins, and play vital roles in mucosal innate immune defense, mucosal repair, and prevention of the infiltration of microorganisms (Hoffmann, 2020). It was also reported that organic acids (mainly containing FA, formate ammonia, propionate, acetate, and lactate) supplementation enhanced duodenal TJPs (including *ZO-1* and *CLDN2*) in broilers (Ma et al., 2021). A compound acidifier blend of calcium formate, calcium citrate and calcium lactate with 7:2:1 ratio could increase the expressions of *MUC2*, *OCLN*, and *CLDN3* in jejunal mucosa of broilers (He et al., 2020). Above all, dietary PFA supplementation could promote small intestinal development through improving intestinal morphology and barrier integrity.

Excessive apoptosis of epithelial cells is an important cause of intestinal mucosal barrier damage (Chen et al., 2021). Caspases are an evolutionary conserved family of cysteine proteases which are centrally involved in inflammation responses and cell death (Abd El-Hack et al., 2022). In the present study, we found that supplementation of 1,000 mg/kg PFA decreased activities of caspase-3 and caspase-8 in small intestinal mucosa. Caspase-8, serving as an initiator caspase, is important player in activation of extrinsic apoptotic pathway (Weng et al., 2014). Caspase-3, an executioner caspase, can be activated by caspase-8 and initiate the process of apoptosis (Van Opdenbosch and Lamkanfi, 2019). Pro-apoptotic Bax and anti-apoptotic Bcl-2 are two important proteins in the Bax family, and upregulation of Bax/Bcl-2 ratio has been proven to increase the activation of caspase-3 and caspase-8 cascade (Du et al., 2014). Consistently, dietary PFA supplementation decreased the expressions of *Bax*, *Bcl-2*, and *Bax*/*Bcl-2* ratio in current study. Therefore, our results of this study suggested that PFA supplementation in the diet could improve intestinal morphology and barrier integrity through suppressing the activation of extrinsic apoptotic pathway in small intestinal mucosa.

Inflammatory response is usually associated with excessive cell apoptosis, leading to the intestinal mucosal barrier damage and gastrointestinal disorders (Van Opdenbosch and Lamkanfi, 2019). It was reported that inflammatory cytokines such as TNF-α, IL-1β, IFN-γ, and IL-6 could induce cell apoptosis (Idriss and Naismith, 2000; Kuo et al., 2021; Ren et al., 2021). In the present study, dietary PFA supplementation decreased concentrations of TNF-α, IL-1β, IL-6, IL-10, and IFN-γ in small intestinal mucosa of broilers. Tumor necrosis factor-alpha, IL-1β, and IFN-γ are three major pro-inflammatory cytokines implicated in the pathogenesis of many inflammatory-associated diseases (Idriss and Naismith, 2000; Jorgovanovic et al., 2020; Kuo et al., 2021). Moreover, TNF-α, IL-1β, and IFN-γ can also activate the generation of pro-inflammatory IL-6 which contributes to the pathogenesis of various diseases, such as inflammation, autoimmunity, and cancers (Suzuki et al., 2000; Raeburn et al., 2002; Ishimaru et al., 2013; Yao et al., 2014). Interleukin-10 is a cytokine with anti-inflammatory properties. Under inflammatory condition, increased IL-6 concentration often accompanied an increased IL-10 concentration (Chun et al., 2007). Therefore, the results of our study indicated that dietary PFA supplementation decreased inflammatory response in small intestinal mucosa of broilers. To further explore the mechanisms underlying the inhibition of intestinal inflammatory response by PFA in broiler chickens, TLR4/NF-κB signaling pathway-related genes expressions were examined in this study. Toll-like receptor 4 is a crucial regulator of inflammatory reactions, whose activation triggers its downstream effector NF-κB, which translocates to the nucleus and upregulates the expressions of pro-inflammatory cytokines such as TNF-α, IL-1β, IL-6, and IFN-γ (Wei et al., 2015; Ye et al., 2021). In the present study, we found that PFA supplementation inhibited the expressions of *TLR4* and *NF-*κ*B* in small intestinal mucosa of broilers. Previous study has demonstrated that encapsulated essential oils and organic acids mixture (containing 4% carvacrol, 4% thyme, 0.5% hexanoic, 3.5% benzoic, and 0.5% butyric acid) supplementation can inhibit necrotic enteritis-induced increase in genes expressions of *TLR4*, *IL-1*β, and *IFN-*γ in the jejunum (Pham et al., 2022). Overall, dietary PFA supplementation could suppress the inflammation response in small intestinal mucosa partially via inhibiting TLR4/NF-κB signaling pathway.

Gut microbiota plays an important role in host gut health by improving gastrointestinal development, enhancing the immune function, and competitively suppressing pathogens (Ley et al., 2008; Camara-Lemarroy et al., 2018; Zhang S. et al., 2021). It has been proven that FA is efficient against pathogenic bacteria through reducing intestinal pH, leading to improvement of gut health in poultry (Hernández et al., 2006; Gharib et al., 2012; Ateya et al., 2019). In the present study, we also found that supplementation of 1,000 mg/kg PFA decreased pH value in cecal digesta of broilers. Besides, PFA supplementation increased the observed species and ACE index of cecal microbiota in broilers. Observed species and ACE index are two important indicators of alpha diversity, and used to calculate unique OTUs and estimate community richness, respectively (Li et al., 2020; Chen et al., 2021). Gut dysbiosis and gastrointestinal inflammatory disease are usually characterized by reduced bacterial richness (Clemente et al., 2018). The results in Song et al. (2022) showed that necrotic enteritis challenge increased intestinal inflammatory cytokine gene expression levels, inhibited intestinal development, and caused intestinal damage, as well as reduced alpha diversity of microbiota in ileum of broilers. Bacteroidetes and Firmicutes were the most predominant phyla in cecal samples of broilers in this study, which was in accord with the results of previous study (Cui et al., 2021). Importantly, dietary PFA supplementation increased the abundance of *Alistipes* that was the dominate genus, and decreased *Methanocorpusculum* abundance in cecal digesta of broilers. Zhang et al. (2022) also found that *Alistipes* was the most plentiful genera in cecum of broilers. *Alistipes* is classified as Gram-negative anaerobic rods, and found primarily in the gut of healthy humans (Shkoporov et al., 2015). Previous study showed that *Alistipes finegoldii* supplementation could decrease the severity of the colitis in mice (Dziarski et al., 2016). Besides, Zhang et al. (2022) reported that dietary rhamnolipids addition could enhance the immunity, improve intestinal barrier function, and increase the cecal abundance of *Alistipes* in broilers. The results suggested that *Alistipes* genus might have a protective role in inflammatory bowel disease. The correlation analysis in this study also showed that the relative abundance of *Alistipes* was negatively correlated with intestinal mucosal concentrations of pro-inflammatory IL-1β and IL-6 which were significantly decreased in PFA group. Previous studies suggesting that *Alistipes* was a short-chain fatty acids (SCFAs) producer (Oliphant and Allen-Vercoe, 2019), which might be contributed to the decreased pH in cecal digesta in this study. Short-chain fatty acids are identified as a principal energy source for intestinal epithelial cells and are known to strengthen the gut barrier function (Parada Venegas et al., 2019). It was reported that SCFAs could lower inflammation and oxidative stress through reducing intestinal permeability and circulating endotoxins (Kim et al., 2018). Besides, SCFAs may signal through cell surface G-protein coupled receptors to activate signaling cascades that regulate immune functions and production of cytokines (Vinolo et al., 2011). *Methanocorpusculum* was the predominant methanogen (Duan et al., 2014), and *Methanocorpusculum* abundance was positively correlated with intestinal mucosal pro-inflammatory TNF-α and IL-6 concentration. The decrease of *Methanocorpusculum* in PFA group might suggested that PFA supplementation benefited to the mitigation of methane emissions in poultry production and the decrease of inflammatory response in intestine of broilers. Therefore, the increased microbial diversity and beneficial bacteria might be another reason for the improved intestinal development in PFA broilers.

## Conclusion

In conclusion, the present study demonstrated that supplementation of 1,000 mg/kg PFA showed beneficial effects in improving intestinal development and function including intestinal morphology, barrier integrity, and nutrients transformation. It might be attributed to the suppression of apoptosis though inhibiting intestinal inflammation partially via inactivating TLR4/NF-κB signaling pathway and change of gut microbiota composition in broiler chickens. These findings will aid in our knowledge of the mechanisms through which dietary PFA modulates gut development, as well as support the use of PFA in poultry industry.

## Data availability statement

The data presented in this study are deposited in the NCBI Sequence Read Archive repository, accession number: PRJNA847797.

## Ethics statement

This animal study was reviewed and approved by the Care and Use Committee of Shandong Agricultural University (protocol code SDAUA-2021-019).

## Author contributions

NJ, WY, and YLi: conceptualization. JL, YLiu, and JN: data curation and project administration. YLiu and CJ: formal analysis. WY and YLi: funding acquisition, supervision, and writing – review and editing. CJ, NJ, LY, and YLi: investigation. YLiu, JN, NJ, LH, SJ, and YLi: methodology. JN, LH, and LY: resources. JL and YLiu: software and writing – original draft. SJ and YLi: validation. CJ and WY: visualization. All authors contributed to the article and approved the submitted version.
