# Supplementary material for: Supplementation with paraformic acid in the diet improved intestinal development through modulating intestinal inflammation and microbiota in broiler chickens
Source: Front Microbiol. 2022 Sep 20;13:975056. doi: 10.3389/fmicb.2022.975056 (PMC9531753; doi:10.3389/fmicb.2022.975056)
Supplement: Supplementary file 1 [file Data_Sheet_1.docx]

**Table S1.** Ingredients composition and nutrient levels of basal diets (as-fed basis).

| Items | Phases | |
| --- | --- | --- |
|  | 0-21 d | 21-42 d |
| Ingredients，% |  |  |
| Corn | 55.91 | 55.91 |
| Soybean meal, 44% CP | 13.78 | 10.18 |
| Wheat bran | 11.98 | 12.98 |
| Corn starch residue | 7.99 | 9.98 |
| Corn gluten meal | 3.99 | 3.99 |
| Extruded soybean | 1.50 | 2.10 |
| Limestone | 1.70 | 1.70 |
| Calcium monophosphate | 1.10 | 1.10 |
| L-Lysine HCl | 1.00 | 1.00 |
| DL-Methionine | 0.20 | 0.20 |
| L-Threonine | 0.10 | 0.10 |
| Sodium chloride | 0.40 | 0.40 |
| Choline | 0.10 | 0.10 |
| Phytase | 0.10 | 0.10 |
| Complex enzyme | 0.02 | 0.02 |
| Trace mineral premix ^1^ | 0.10 | 0.10 |
| Vitamin premix ^2^ | 0.02 | 0.02 |
| Antioxidant | 0.02 | 0.02 |
| Total | 100 | 100 |
| Calculated analysis，% |  |  |
| Metabolizable energy，MJ/kg | 12.33 | 12.50 |
| Crude protein | 19.47 | 17.93 |
| Crude fat | 3.45 | 3.74 |
| Calcium，% | 0.94 | 0.87 |
| Available phosphorus，% | 0.35 | 0.33 |
| Lysine，% | 1.15 | 1.00 |
| Methionine，% | 0.50 | 0.40 |

^1^ Provided per kilogram of complete basal diet: 10 mg of Cu as CuSO_4_, 100 mg of Fe as FeSO_4_, 1.1 mg of I as Ca(IO_3_)_2_, 65 mg of Zn as ZnSO_4_, 100 mg of Mn as MnSO_4_ and 0.3 mg of Se as Na_2_SeO_3_.

^2^ Provided per kilogram of complete basal diet: vitamin A 10,000 IU, vitamin D_3_ 3,000 IU, vitamin E 30 IU, vitamin K_3_ 1.3 mg, vitamin B_1_ 2.2 mg, vitamin B_2_ 8 mg, vitamin B_3_ 8 mg, vitamin B_6_ 4 mg, vitamin B_12_ 0.025 mg, biotin 0.2 mg, niacin 40 mg, folic acid 1 mg and D-calcium pantothenate 10 mg.

**Table S2.** Primer sequences used for quantitative real-time PCR.

| Genes | Gene bank No. | Primer sequences ^a^ (5’-3’) |
| --- | --- | --- |
| *β-actin* | NM_205518.1 | F: TTGGTTTGTCAAGCAAGCGG |
|  |  | R: CCCCCACATACTGGCACTTT |
| *OCLN* | NM_205128.1 | F: ACGGCAGCACCTACCTCAA |
|  |  | R: GGGCGAAGAAGCAGATGAG |
| *CLDN2* | NM_001277622.1 | F: CTGCTCACCCTCATTGGA |
|  |  | R: AACTCACTCTTGGGCTTCTG |
| *CLDN3* | XM_204202.1 | F: CCCGTCCCGTTGTTGTTTTG |
|  |  | R: CCCCTTCAACCTTCCCGAAA |
| *ZO-1* | XM_015278975.2 | F: CTTCAGGTGTTTCTCTTCCTCCTC |
|  |  | R: CTGTGGTTTCATGGCTGGATC |
| *GLUT2* | XM_010716927.3 | F: TCATTGTAGCTGAGCTGTT |
|  |  | R: TTGCTGGCTTTGGGTTGTG |
| *SGLT1* | NM_001293240.1 | F: TGTCTCTCTGGCAAGAACATGTC |
|  |  | R: GGGCAAGAGCTTCAGGTATCC |
| *y+LAT1* | XM_040665181.1 | F: CAGAAAACCTCAGAGCTCCCTTT |
|  |  | R: TGAGTACAGAGCCAGCGCAAT |
| *FABP1* | NM_204192 | F: ACTGGCTCCAAAGAATGACCAATG |
|  |  | R: TGTCTCCGTTGAGTTCGGTCAC |
| *CAT1* | XM_015277945.1 | F: CAAGAGGAAAACTCCAGTAATTGCA |
|  |  | R: AAGTCGAAGAGGAAGGCCATAA |
| *TLR4* | NM_001030693.1 | F: AGGCACCTGAGCTTTTCCTC |
|  |  | R: TACCAACGTGAGGTTGAGCC |
| *NF-κB* | NM_001396038.1 | F: CAGCCCATCTATGACAACCG |
|  |  | R: TCAGCCCAGAAACGAACCTC |
| *Bax* | XM_422067 | F: GGTGACAGGGATCGTCACAG |
|  |  | R: TAGGCCAGGAACAGGGTGAAG |
| *Bcl-2* | NM_205339.2 | F: GCTGCTTTACTCTTGGGGGT |
|  |  | R: CTTCAGCACTATCTCGCGGT |

^a^ F, forward; R, reverse.

**Table S3.** Operational taxonomic unit (OUT) clustering and annotation per sample.

| **Samples^1^** | **Total tags** | **Taxon tags** | **Unclassified tags** | **Unique tags** | **OUT numbers** |
| --- | --- | --- | --- | --- | --- |
| CON1 | 69175 | 65229 | 1 | 3945 | 807 |
| CON2 | 62805 | 58701 | 0 | 4104 | 945 |
| CON3 | 52404 | 47362 | 0 | 5042 | 881 |
| CON4 | 64690 | 61068 | 0 | 3622 | 854 |
| CON5 | 57035 | 52496 | 2 | 4537 | 827 |
| CON6 | 57563 | 53749 | 0 | 3814 | 801 |
| CON7 | 62469 | 59818 | 5 | 2646 | 797 |
| PFA1 | 62655 | 59023 | 2 | 3630 | 744 |
| PFA2 | 66116 | 62399 | 0 | 3717 | 849 |
| PFA3 | 57595 | 53500 | 0 | 4095 | 938 |
| PFA4 | 43398 | 38712 | 0 | 4686 | 952 |
| PFA5 | 61231 | 56707 | 0 | 4524 | 938 |
| PFA6 | 64045 | 59829 | 3 | 4213 | 861 |
| PFA7 | 53852 | 49698 | 0 | 4154 | 953 |

^1^CON 1, 2, 3, 4, 5, 6, and 7 means cecal digesta samples from broilers fed with a basal diet; PFA 1, 2, 3, 4, 5, 6, and 7 means cecal digesta samples from broilers fed a basal diet supplemented with 1,000 mg/kg paraformic acid.

**Table S4.** Effects of dietary paraformic acid supplementation on the relative abundance of cecal microbiota at the phylum level.

| **Items, %** | **Treatment ^1^** | | ***p* value** |
| --- | --- | --- | --- |
|  | **CON** | **PFA** |  |
| Bacteroidota | 31.62±4.99 | 35.71±3.72 | 0.525 |
| Firmicutes | 38.08±3.20 | 36.95±4.23 | 0.807 |
| Euryarchaeota | 0.03±0.01 | 3.30±3.22 | 0.329 |
| Desulfobacterota | 7.23±1.73 | 9.40±1.58 | 0.373 |
| Halobacterota | 8.02±1.92 | 2.70±0.74 | 0.033 |
| Synergistota | 1.77±0.48 | 2.44±1.05 | 0.572 |
| Verrucomicrobiota | 1.26±1.16 | 0.32±0.12 | 0.436 |
| Campylobacterota | 3.08±1.02 | 0.72±0.13 | 0.060 |
| Proteobacteria | 1.07±0.69 | 0.82±0.40 | 0.766 |
| unidentified_Bacteria | 2.16±0.25 | 2.69±0.39 | 0.273 |

Values are mean ± standard error (*n* = 7). Differences were considered statistically significant when *P* < 0.05.

^1^ CON, broilers fed a basal diet; PFA, broilers fed a basal diet supplemented with 1,000 mg/kg paraformic acid.

**Table S5.** Effects of dietary paraformic acid supplementation on the relative abundance of cecal microbiota at the genus level (excluding *Alistipes* and *Methanocorpusculum*).

| **Items, %** | **Treatment ^1^** | | ***p* value** |
| --- | --- | --- | --- |
|  | **CON** | **PFA** |  |
| *Bacteroides* | 10.91±4.91 | 6.09±2.82 | 0.412 |
| *Methanobrevibacter* | 0.02±0.01 | 3.30±3.22 | 0.348 |
| *Desulfovibrio* | 6.70±1.68 | 8.99±1.55 | 0.335 |
| *Megamonas* | 1.74±0.68 | 2.44±1.89 | 0.734 |
| *Faecalibacterium* | 5.59±1.58 | 5.25±1.48 | 0.877 |
| *CHKCI001* | 2.42±1.53 | 1.54±0.36 | 0.589 |
| *[Ruminococcus]_torques_group* | 3.48±0.77 | 3.83±1.11 | 0.804 |
| *Synergistes* | 1.77±0.48 | 2.44±1.05 | 0.572 |
| *Akkermansia* | 1.25±1.16 | 0.29±0.12 | 0.425 |
| *Barnesiella* | 3.00±0.71 | 3.56±0.89 | 0.631 |
| *Helicobacter* | 3.06±1.02 | 0.71±0.14 | 0.060 |
| *Parabacteroides* | 1.47±0.41 | 1.65±0.69 | 0.825 |
| *Prevotellaceae_UCG-001* | 1.03±0.52 | 1.11±0.60 | 0.921 |
| *Ligilactobacillus* | 0.37±0.15 | 0.80±0.56 | 0.463 |
| *Phascolarctobacterium* | 2.09±0.34 | 2.14±0.20 | 0.902 |
| *Pseudomonas* | 0.45±0.45 | 0.28±0.28 | 0.744 |
| *Limosilactobacillus* | 0.07±0.03 | 0.38±0.32 | 0.380 |
| *Romboutsia* | 1.43±0.15 | 1.02±0.19 | 0.122 |
| *NK4A214_group* | 0.89±0.20 | 0.49±0.07 | 0.103 |
| *Olsenella* | 0.02±0.01 | 0.27±0.22 | 0.308 |
| *Serratia* | 0.23±0.23 | 0.14±0.14 | 0.745 |
| *Lactobacillus* | 0.10±0.02 | 0.24±0.19 | 0.491 |
| *unidentified_Erysipelotrichaceae* | 0.31±0.08 | 0.28±0.18 | 0.914 |
| *Christensenellaceae_R-7_group* | 0.80±0.12 | 0.54±0.05 | 0.082 |
| *Butyricicoccus* | 0.50±0.12 | 0.64±0.15 | 0.468 |
| *Colidextribacter* | 0.83±0.10 | 0.66±0.07 | 0.211 |
| *Erysipelatoclostridium* | 0.57±0.12 | 0.53±0.12 | 0.835 |
| *Ruminococcus* | 0.49±0.08 | 0.33±0.04 | 0.077 |
| *Bilophila* | 0.43±0.07 | 0.29±0.05 | 0.106 |
| *Subdoligranulum* | 0.23±0.08 | 0.11±0.03 | 0.169 |
| *Fusobacterium* | 0.14±0.08 | 0.06±0.04 | 0.413 |
| *UCG-005* | 0.25±0.06 | 0.15±0.03 | 0.174 |
| *Cerasicoccus* | 0.00±0.00 | 0.02±0.02 | 0.339 |

Values are mean ± standard error (*n* = 7). Differences were considered statistically significant when *P* < 0.05.

^1^ CON, broilers fed a basal diet; PFA, broilers fed a basal diet supplemented with 1,000 mg/kg paraformic acid.


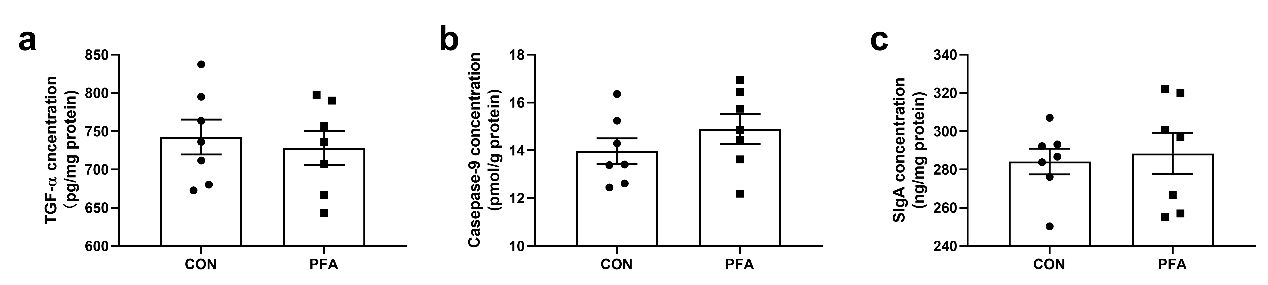


**Figure S1.** Effects of dietary paraformic acid supplementation on intestinal mucosal barrier functions, apoptosis regulators, and inflammatory factors concentrations. (**a**) Transforming growth factor-α (TGF-α); (**b**) Caspase-9; (**c**) Secretory immunoglobulin A (SIgA). CON, broiler chickens fed basal diet; PFA, broiler chickens fed basal diet supplemented with 1,000 mg/kg paraformic acid. Values are mean ± standard error (*n* = 7).


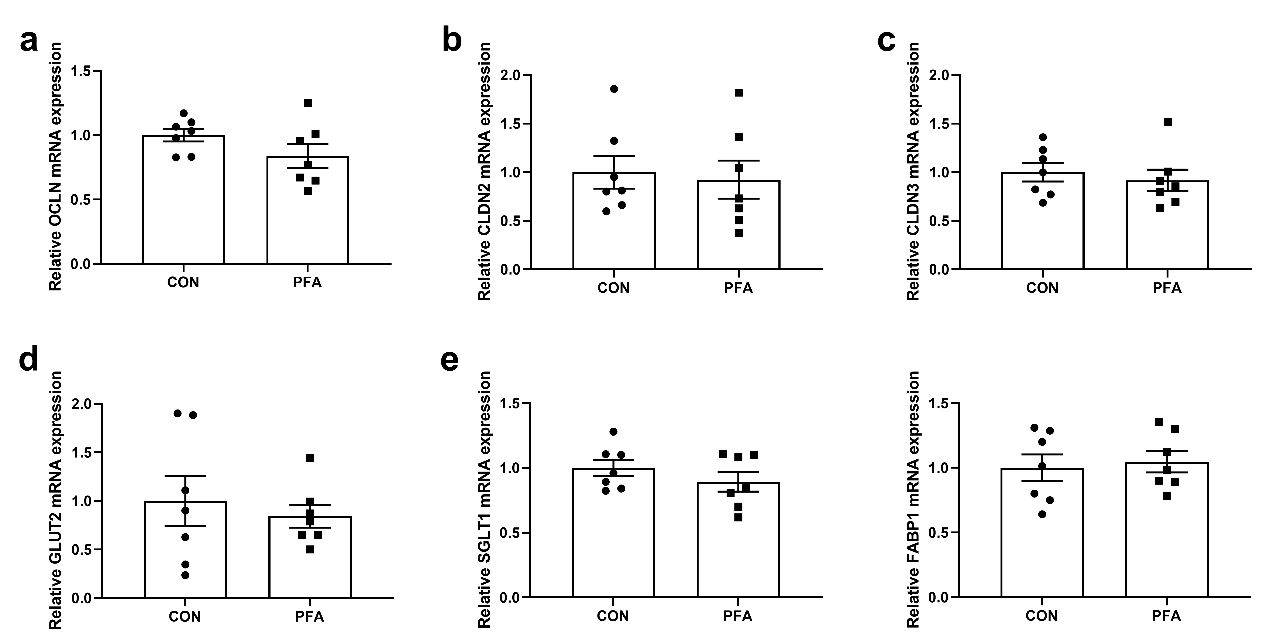


**Figure S2.** Effect of dietary paraformic acid supplementation on relative mRNA expression in intestinal mucosa of broilers. (**a**) Occluding (OCLN); (**b**) Claudin 2 (CLDN2); (**c**) Claudin 3 (CLDN3); (**d**) Glucose transporter 2 (GLUT2); (**e**) Na+/glucose cotransporter (SGLT1); (**f**) Fatty acid binding protein (FABP1). CON, broiler chickens fed basal diet; PFA, broiler chickens fed basal diet supplemented with 1,000 mg/kg paraformic acid. Values are mean ± standard error (*n* = 7).


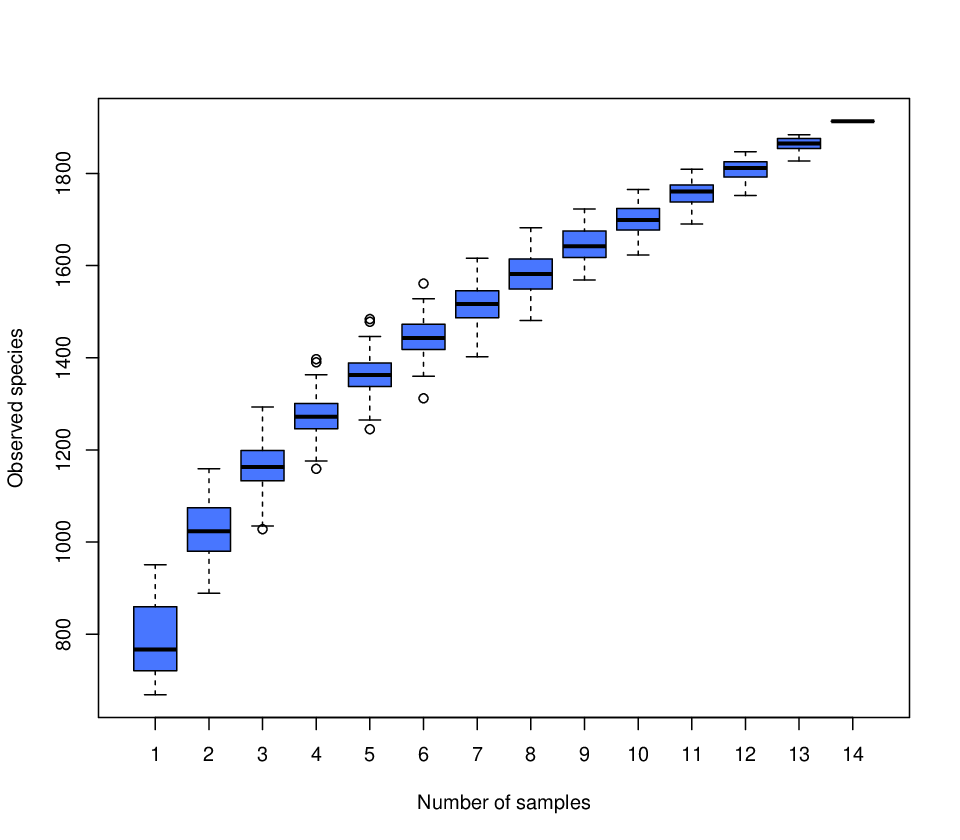


**Figure S3.** Species accumulation curves (SAC). The SAC tends to flatten with analyzed sequences number increasing up to 14, demonstrating that our samples were sufficient for OTU testing and prediction of species richness of samples.


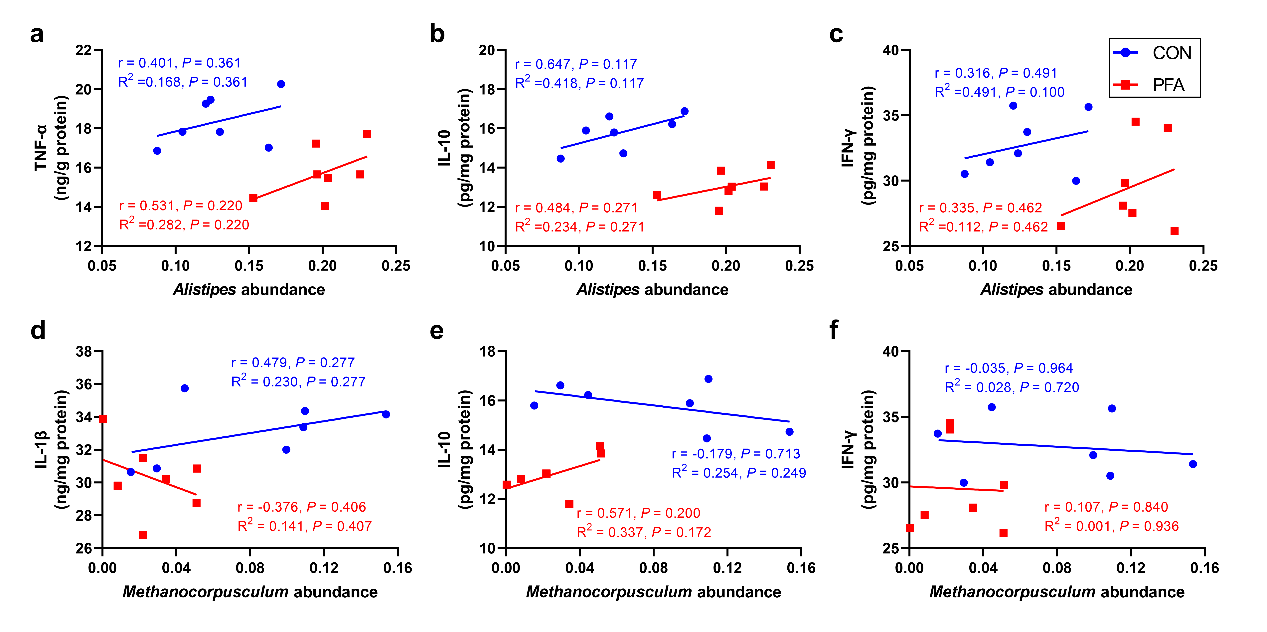


**Figure S4.** Correlation analysis between *Alistipes* and *Methanocorpusculum* abundances and mucosal immunological markers concentrations. (**a-c**) Correlation analysis between *Alistipes* abundance and mucosal concentrations of tumor necrosis factor-alpha (TNF-α), interleukin-10 (IL-10), and interferon‐γ (IFN‐γ) for CON group and PFA group; (**d-f**) Correlation analysis between *Methanocorpusculum* abundance and mucosal concentrations of interleukin-1beta (IL-1β), IL-10, and IFN‐γ for CON group and PFA group. Correlation analysis was based on Spearman’s test and simple linear regression. Statistical significance was set at *P* < 0.05. *n* = 7.
